# Comparative Analysis of Single-Channel and Multi-Channel Classification of Sleep Stages Across Four Different Data Sets

**DOI:** 10.3390/brainsci14121201

**Published:** 2024-11-28

**Authors:** Xingjian Zhang, Gewen He, Tingyu Shang, Fangfang Fan

**Affiliations:** 1 Academy of Medical Engineering and Translational Medicine, Tianjin University, Tianjin 300072, China; 2019235005@tju.edu.cn; 2 Department of Computer Science, Florida State University, Tallahassee, FL 32306, USA; he@cs.fsu.edu; 3 School of Mathematics and Statistics, Shaanxi Normal University, Xi’an 710062, China; 20241692@snnu.edu.cn; 4 Department of Neurology, Beth Isreal Deaconess Medical Center, Harvard Medical School, Harvard University, Cambridge, MA 02215, USA

**Keywords:** automatic sleep stage classification, multi-channel signals, multi-data set analysis, multi-class tasks, deep learning, hybrid attention neural network

## Abstract

**Background**: Manually labeling sleep stages is time-consuming and labor-intensive, making automatic sleep staging methods crucial for practical sleep monitoring. While both single- and multi-channel data are commonly used in automatic sleep staging, limited research has adequately investigated the differences in their effectiveness. **Methods:** In this study, four public data sets—Sleep-SC, APPLES, SHHS1, and MrOS1—are utilized, and an advanced hybrid attention neural network composed of a multi-branch convolutional neural network and the multi-head attention mechanism is employed for automatic sleep staging. **Results**: The experimental results show that, for sleep staging using 2–5 classes, a combination of single-channel electroencephalography (EEG) and dual-channel electrooculography (EOG) consistently outperforms single-channel EEG with single-channel EOG, which in turn outperforms single-channel EEG or single-channel EOG alone. For instance, for five-class sleep staging using the MrOS1 data set, the combination of single-channel EEG and dual-channel EOG resulted in an accuracy of 87.18%, whereas the combination of single-channel EEG and single-channel EOG yielded an accuracy of 85.77%. In comparison, single-channel EEG alone achieved an accuracy of 85.25% and single-channel EOG alone achieved an accuracy of 83.66%. **Conclusions**: This study highlights the significance of combining EEG and EOG signals in automatic sleep staging, while also providing valuable insights for the channel design of portable sleep monitoring devices.

## 1. Introduction

Sleep is one of the most important fundamental processes of life, being crucial for individual health and well-being [1]. The cumulative effects of poor sleep quality are associated with serious health problems such as cardiovascular diseases, depression, and heart attack [2]. Nevertheless, a significant number of people worldwide experience sleep problems regardless of age, demographic, or health status [3]. For instance, in the United States, approximately 70% of adults report poor sleep quality at least once a month [4].

To solve sleep problems effectively, it is necessary to monitor sleep quality through sleep staging [5]. At present, overnight polysomnography (PSG) is considered the gold standard for evaluating sleep stages [6]. Sleep experts follow widely accepted guidelines for sleep scoring, such as those from the American Academy of Sleep Medicine (AASM) [7] or Rechtschaffen and Kales (R&K) [8]. According to the AASM manual, PSG signals are segmented into 30 s epochs, and sleep activities are classified into five categories: wake (when the person is fully awake and alert); non-rapid eye movement stage I (N1, the lightest stage of sleep where one can be easily awakened); non-rapid eye movement stage II (N2, a slightly deeper stage of sleep with slower brain waves and occasional bursts of rapid brain activity); non-rapid eye movement stage III (N3, the deepest stage of NREM sleep, also known as slow-wave sleep, crucial for physical restoration and growth); and rapid eye movement (REM, where most dreaming occurs and the brain is highly active while the body is in a state of temporary paralysis). The R&K manual further divides N3 into the N3 and non-rapid eye movement stage IV (N4) stages. In practical applications, a variety of multi-class sleep staging standards are implemented [9]. For instance, the two-class approach designates sleep stages as Wake and Sleep, whereas the three-class standard distinguishes Wake, non-rapid eye movement (NREM), and REM, and the four-class category divides the stages into Wake, Light sleep (Light), Deep sleep (Deep), and REM. At present, the AASM manual is more commonly used. However, manual sleep staging by sleep experts is both time-consuming and expensive, with results often affected by subjective bias. Therefore, with the development of deep learning, many researchers have utilized this technology for automatic sleep staging [10,11].

Automatic sleep staging methods generally involve single- and multi-channel data. Regarding single-channel approaches, Supratak et al. [12] utilized single-channel electroencephalography (EEG) data and proposed DeepSleepNet. This model combines a convolutional neural network (CNN) with a bidirectional long short-term memory network (Bi-LSTM), achieving accuracies between 76.9% and 86.2% on two public data sets. Similarly, Mousavi et al. [13] leveraged single-channel EEG data from the same public data sets, achieving sleep staging accuracies ranging from 77.6% to 84.3%. Fan et al. [14] employed single-channel electrooculography (EOG) data for sleep staging, achieving overall accuracies of 81.2% and 76.3% with a hybrid CNN and gated recurrent unit (GRU) model. Wei et al. [15] investigated sleep staging with single-channel electrocardiogram (ECG) data from their own private data sets, obtaining an accuracy of 71.16% using a simple LSTM model.

Regarding the latter category, Toma et al. [16] achieved a sleep staging accuracy of 90.21% using a CNN–Bi-LSTM model based on multi-channel data, incorporating two EEG channels, one EOG channel, and one electromyogram (EMG) channel. Yao et al. [17] employed a novel method for sleep staging by converting data from six EEG channels and two EOG channels into time-frequency images. They then constructed a model using the spatial–temporal transformer network based on the Vision Transformer architecture. This approach yielded accuracies of 82.9%, 84%, and 89.2% on three public data sets. Pathak et al. [18] utilized data from two EEG channels, two EOG channels, and one EMG channel and constructed a CNN–Bi-LSTM model with residual connections, achieving an accuracy of 85%.

However, despite the potential benefits of multi-channel data in automatic sleep staging, there has been insufficient research on a comprehensive comparison of their effectiveness with respect to single-channel data. Lu et al. [19] conducted a study that showed the advantages of multi-channel data in sleep staging. However, there are some concerns with their methodology. Firstly, the evaluation methods used for single- and multi-channel data experiments were different. Additionally, there were significant differences in the model architectures used for single- and multi-channel data. This raises the possibility that the observed advantages of multi-channel data might be due to the different evaluation methods or the use of more advanced models. Similarly, in [20], Siddiqa et al. compared the performance of single- and multi-channel EEG data. However, they focused solely on combining multiple EEG signals, overlooking the EOG signals, which are crucial for providing additional valuable information in sleep staging. In another study, Paisarnsrisomsuk et al. [21] demonstrated that adding an EOG channel to the existing EEG channels increased the sleep staging accuracy by only 1%. However, their study only involved a basic CNN architecture, which likely did not fully exploit the multi-channel data. Additionally, as their study only used the Sleep-EDF data set, the small sample size may have limited the generalizability of their conclusions.

Given the three limitations mentioned above, we performed a more comprehensive study to investigate the differences in the effectiveness of single- and multi-channel data in automatic sleep staging. First, we utilized four public data sets, namely Sleep-SC (https://physionet.org/content/sleep-edfx/1.0.0/, accessed on 5 November 2023), APPLES (https://sleepdata.org/datasets/apples, accessed on 14 November 2023), SHHS1 (https://sleepdata.org/datasets/shhs, accessed on 19 November 2023), and MrOS1 (https://sleepdata.org/datasets/mros, accessed on 24 November 2023), each with different characteristics, thus enhancing the generalizability of the conclusions. For example, one data set consisted solely of healthy individuals, while another mostly included participants with obstructive sleep apnea (OSA). Second, to fully exploit and harness the sleep data, we employed an advanced hybrid attention neural network comprising a multi-branch CNN and the multi-head attention mechanism of the Transformer model. Third, we conducted experiments under various application scenarios using a multi-task setting, including sleep staging classification using 2–5 categories, in order to investigate whether different tasks yielded different conclusions. The key contributions of this work are as follows:

(1) Four public data sets with different characteristics—namely, Sleep-SC, APPLES, SHHS1, and MrOS1—were evaluated for single- and multi-channel automatic sleep staging to obtain more generalizable conclusions.
(2) Experiments were conducted across various sleep staging tasks, specifically involving 2-class, 3-class, 4-class, and 5-class sleep staging classification. This approach was used to assess the consistency of outcomes across different classification tasks.
(3) An advanced hybrid attention neural network was employed to fully exploit and harness the information from the sleep data.
(4) The findings highlight the significance of combining EEG and EOG signals in automatic sleep staging, while also providing valuable insights for the channel design of portable sleep monitoring devices.

The remainder of this study is organized as follows: In Section 2, we describe the four evaluation data sets and the automatic sleep staging framework in detail. In Section 3, we compare the evaluation results of multi-channel data with those of single-channel data across four tasks, with particular emphasis on the five-class designation. In Section 4, the experimental results are discussed. Section 5 provides the study’s conclusion.

## 2. Materials and Methods

This chapter has six sections. Section A, “Data sets”, describes the four public data sets we used. Section B, “Data pre-processing”, details how we cleaned and prepared the data for sleep staging model. Section C, “Sleep Staging Model Architecture”, outlines the design of our sleep staging model, describing its structure and layers. Section D, “Other Classification Models”, introduces other models we used for comparison. Section E, “Experimental Strategy”, explains our experimental design. Finally, Section F, “Performance Metrics”, describes the metrics we used to evaluate our model.

A. Data sets

The Sleep-EDF data set is perhaps the most commonly used for evaluating sleep staging algorithms and includes two different recording methods: sleep telemetry (ST) [22] and sleep cassette (SC) [23]. Due to the small sample size of the ST data set, which includes data from only 22 individuals, this experiment utilized the SC data set, referred to as Sleep-SC. Sleep-SC comprises 153 PSG files collected from 78 individuals. Each subject’s PSG data were recorded over two nights, with a total of three nights of data missing. Data were collected between 1987 and 1991, and the subjects were healthy Caucasians aged 25–101, without any sleep-related medication. The PSG data primarily include EEG, EOG, and EMG signals. The EEG signals (from Fpz-Cz and Pz-Oz channels) were sampled at 100 Hz, the horizontal EOG signal was also sampled at 100 Hz, and the EMG signal was sampled at 1 Hz.

The APPLES data set originates from The Apnea Positive Pressure Long-term Efficacy Study [24], a multi-center trial that started in November 2003 and lasted for six months, which was conducted in five university hospitals or private clinics in the United States. A total of 1516 subjects participated, most of whom were OSA patients, resulting in 1104 PSG recordings in the APPLES data set. Each PSG recording includes 4 EEG channels and 2 EOG channels, as well as EMG, ECG, and other channels. The sampling rate for both EEG and EOG signals is 100 Hz. Despite its potential challenges, particularly in sleep staging for OSA patients, we chose this data set to objectively compare the performance of single- and multi-channel data in sleep staging, due to its distinctive features.

The Sleep Heart Health Study (SHHS) is a multi-center cohort study exploring the effects of sleep-disordered breathing on cardiovascular health [25]. SHHS Visit 1, referred to as SHHS1 in our research, was conducted between November 1995 and January 1998. The results include PSG data from 6441 participants aged 40 or older, most of whom had diseases such as coronary heart disease, stroke, or hypertension, resulting in 5681 PSG files. Each PSG recording includes data from 2 EEG channels and 2 EOG channels, as well as EMG, ECG, and other channels. The sampling rate is 125 Hz for EEG and 50 Hz for EOG signals. As a large-scale sleep study, SHHS1 has been widely used in previous research, and serves as a benchmark for comparison in this study.

The MrOS is an ancillary study considering osteoporotic fractures in men [26]. MrOS Visit1, referred to as MrOS1 in our research, was conducted between 2000 and 2002. PSG data were collected from 5994 community-dwelling men aged 65 years or older, most of whom had sleep disorders, osteoporosis, and vascular diseases, resulting in 2907 PSG files. Each PSG recording includes data from 4 EEG channels (unreferenced, with only 2 EEG channels after re-referencing) and 2 EOG channels, as well as EMG, ECG, and other channels. The sampling rate for EEG and EOG signals is 512 Hz. The MrOS1 data set is notable for its focus on elderly males with sleep disorders; therefore, it was used as one of the evaluation data sets in this study.

The Sleep-SC data set is available for direct download from the PhysioNet database [27]. The other three data sets are available upon request and only after obtaining authorization from the NSRR [28]. The key information of the four public data sets is summarized in Table 1. In addition, these data sets were used in compliance with the terms and conditions set by their providers. No additional ethical approval was required for this study, as the data had already been de-identified and were publicly available.

B. Data pre-processing

To ensure fair comparisons between different data sets and streamline the experimental process, we selected 500 PSG records from the beginning of each data set, except for Sleep-SC, which had 153 PSG records. Only PSG records with complete data were chosen, excluding any with missing channels or signal loss. Consequently, in the Sleep-SC data set, Record 4362 was excluded due to the signal being shorter than the label, indicating signal loss. In the MrOS1 data set, Records 0237, 0402, and 0641 were excluded for the same reason. Additionally, 12 records in the APPLES data set (240043, 240044, 240047, 240048, 240049, 240051, 240052, 240053, 240054, 240056, 330002, and 330004) and 16 records in the SHHS1 data set (200144, 200155, 200197, 200202, 200312, 200382, 200383, 200398, 200410, 200469, 200498, 200555, 200562, 200579, 200630, and 200632) were excluded due to missing channels.

Following PSG record selection, the Python-MME library [29] was used for data pre-processing. Initially, signal (.edf) and label files were merged along the time dimension, eliminating any data without corresponding labels. Subsequently, the sampling rate of the data was standardized to 100 Hz, followed by notch filtering and band-pass filtering between 1 and 40 Hz. The aim of the notch filtering was to eliminate power line interference from the data. Since the Sleep-SC data set was collected in Europe, we applied a notch filter at 50 Hz. For the other three data sets, which were collected in the United States, we applied a notch filter at 60 Hz. The primary purpose of the band-pass filtering was to remove noise interference, with high-energy noise predominantly concentrated below 1 Hz. Therefore, we set the lower cut-off frequency at 1 Hz to effectively filter out this noise while still capturing the majority of the delta wave activity. This approach helps to balance noise elimination with the preservation of relevant sleep stage information. Specifically, for the MrOS1 data set, channels C3 and C4 were re-referenced to M1 and M2, resulting in C3-M2 and C4-M1. The processed files were then segmented into 30 s epochs, with any incomplete epochs at the end of the files removed. Finally, if Stage N4 was present, N3 and N4 were merged into N3 to comply with the AASM manual. Additionally, epochs irrelevant to sleep staging (i.e., aside from Wake/N1/N2/N3/REM) were excluded. This process resulted in a substantial number of epochs for each data set, as detailed in Table 2, with each epoch serving as a training and testing sample for the sleep staging algorithm.

**Figure 1:**
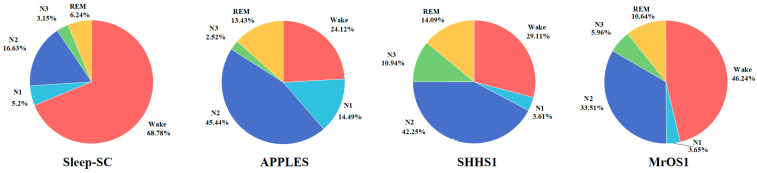
Distribution ratios of different sleep stages in each data set. Red represents Wake, light blue represents N1, dark blue represents N2, green represents N3, and yellow represents REM.

The distribution ratios of different sleep stages are depicted in Figure 1, revealing a disproportionate distribution of epoch categories within each data set, predominantly encompassing the Wake, N2, and REM stages.

C. Architecture of the sleep staging model

**Figure 2:**
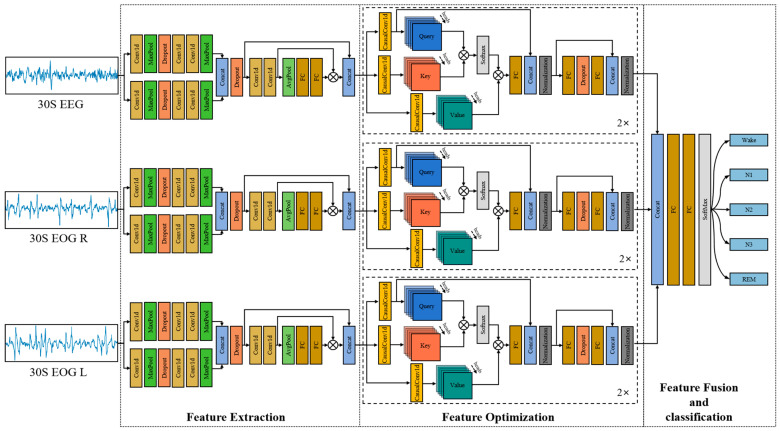
The architecture of sleep staging model using multi-channel data comprises three modules for feature extraction and optimization, as well as feature fusion and classification. Each layer comprises a one-dimensional convolutional layer (Conv1d), a max-pooling layer (Maxpool), a dropout layer (Dropout), a concatenation layer (Concat), a global average pooling layer (Avgpool), a fully connected layer (FC), a causal convolutional layer (CausalConv1d), and a normalization layer (Normalization). Additionally, ‘2×’ indicates that the structure within the dashed box is repeated twice, and 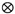 refers to point-wise multiplication.

The architecture of the sleep staging model based on the hybrid attention neural network is illustrated in Figure 2.

Inspired by Eldele et al. [30], we divided the hybrid attention neural network into feature extraction, feature optimization, feature fusion, and classification modules. Initially, data from each channel were processed independently with the feature extraction and feature optimization modules to obtain highly abstracted and optimized features. For instance, if there were three data channels, three sets of feature extraction and feature optimization modules were used, as shown in Figure 2. Similarly, if using the single-channel data, only one set of these modules was considered. Then, the highly abstracted and optimized features were fused and classified. For simplicity in describing the architecture, we only consider the case with the single-channel data used as input.

In the feature extraction module, two different convolutional kernel sizes were employed in parallel CNN branches. This multi-branch design approach, originally introduced in GoogLeNet [31], allows for the extraction of features at different scales. Specifically, the first branch’s initial Conv1D layer had 64 kernels with a size of 50 and a stride of 6, followed by max-pooling layers with kernel sizes of 8 and 4, and respective strides of 2 and 4. The second branch’s initial Conv1D layer has 64 kernels with a size of 400 and a stride of 5, followed by max-pooling layers with kernel sizes of 4 and 2 and strides of 2. All other Conv1D layers had 128 kernels with a size of 7 and a stride of 1. The dropout rate was set at 0.5. Then, further feature extraction was performed using a CNN with a channel attention mechanism. This channel attention mechanism, first proposed in SE-ResNet [32], generates channel weights through global average pooling and FC layers, adjusting the feature intensity to focus on more relevant sleep staging features. Additionally, utilizing the shortcut method of ResNet [33] allows for more efficient feature transmission. Specifically, the two Conv1D layers both had 30 kernels with a size of 1 and a stride of 1. The first FC layer had 2 neurons, and the second had 30.

In the feature optimization module, the extracted features were transformed into three vector spaces through causal convolutional layers. The causal convolutional layer, first introduced in the temporal convolutional network (TCN) [34], ensures that convolution at a specific time step is only dependent on the inputs at previous time steps. This is crucial for sleep staging tasks involving sequential data, where future information should not be used to predict past sleep stages. The CausalConv1d layer had 30 kernels with a size of 7 and a stride of 1. Then, imitating the multi-head attention mechanism (MHA) in the Transformer model [35], the three generated vector spaces—referred to as Query, Key, and Value—interact through scaled dot-product attention, enabling the model to focus on the most relevant parts and capture different aspects of the input sequence. Additionally, the input features were divided into subspaces and their attention weights were concatenated, enhancing the overall representation of their importance. By leveraging this attention mechanism, the model can effectively manage the complexities of sleep staging, enhancing its ability to accurately classify different sleep stages. The formula of the multi-head attention mechanism is as follows:

(1) $$\;Attention\left(Q,\;K,\;V\right)=softmax\left(\frac{Q{\;K}^{T}\;}{\sqrt{d}}\right)\;V\;$$

where *Q*, *K*, and *V* refer to Query, Key, and Value, respectively, and *d* refers to the number of subspaces (specifically, the value of *d* was set to 5 in this study). The first FC layer had 80 neurons, the second had 120 neurons with a dropout rate set to 10%, and the third had 80 neurons in the feature optimization module.

In the feature fusion and classification module, the optimized features were first merged into a larger feature vector along the feature dimension. This larger feature vector was then processed through two FC layers. The first FC layer had 80 neurons, and the second had a number of neurons equal to the number of sleep stages, followed by the SoftMax function for sleep stage classification.

D. Other classification models for comparison

To comprehensively compare the sleep staging performance differences between single-channel and multi-channel data, both machine learning models and other deep learning models were employed for comparison.

For the machine learning models, two models were selected: Support Vector Machine (SVM) and Random Forest (RF), sourced from the Scikit-learn library [36]. For SVM, the radial basis function (RBF) kernel was used, with the regularization parameter set to 1. For RF, the Gini impurity was used as the measure of split quality, and the number of trees was set to 100. Since machine learning models cannot automatically extract features as deep learning models do, we manually extracted commonly used features from the time domain, frequency domain, and nonlinear domain. In the time domain, the mean and standard deviation were calculated; in the frequency domain, the spectral energy and dominant frequency were calculated; and in the nonlinear domain, Higuchi’s fractal dimension and Lempel-Ziv Complexity were calculated. Thus, each 30 s sample generated six features.

For the other deep learning models, two models were selected: EEGNet [37] and a simplified sleep staging model. EEGNet features depthwise and separable convolutions, which have proven effective in sleep EEG classification [38]. The simplified sleep staging model was used for ablation analysis; its architecture is similar to the model introduced in subsection C, but without the feature optimization modules.

E. Experimental Strategy

To overcome the potential bias associated with sample selection, we followed the methodology described in [39]. Five-fold cross-validation was carried out to evaluate the performance of the automatic sleep staging method. To ensure the independence between training and testing, data were divided into five folds, each comprising PSG records as the smallest unit, rather than epochs. This approach prevented data from the same night from being split between training and testing sets, thereby enhancing the model’s generalizability to real-world scenarios.

For fair comparison between single- and multi-channel data, identical parameters were used for all sleep staging classification tasks. When training the deep neural network, the Adam [40] optimizer was employed with a learning rate of 0.001 and a weight decay of 0.001, with the AMSGrad [41] mechanism enabled. To ensure optimal learning dynamics during training, a learning rate scheduler was employed with a step size of 10 epochs and a decay factor (gamma) of 0.5. Specifically, regardless of whether machine learning or deep learning was used, the weighted cross-entropy loss function was applied to address the disproportionate distribution of epochs across different sleep stages. The weights were calculated as follows:

(2) $$max_{num}=max\left(labels_{num\left[k\right]}\right),\;\;k\in classes$$

(3) $$class\_weight[k]=\sqrt{\frac{max\_num}{labels\_num[k]}\;}$$

Considering that the N1 stage is particularly difficult to classify [42], its initial weight was set to 1.5 times that of the other stages. To prevent significant discrepancies in class weights affecting the loss calculation, a threshold was set in which no class weight exceeded 3.5 times that of any other class. The weighted cross-entropy loss function ensures that the model focuses more on minority sleep stage classes through adjusting the loss values according to class weights.

The experiment was conducted using Python 3.8.13, Scikit-learn 0.24.2 and PyTorch 2.3.0 in a CentOS Stream 8 environment. The hardware used for training and testing included a 12th Gen Intel Core i7-12700KF CPU, a GeForce RTX 3090 GPU, and 64 GB of RAM.

F. Performance metrics

The performance of the model using single- and multi-channel data was evaluated in terms of the overall accuracy (Acc), Cohen’s Kappa coefficient (κ), and per-class F1 score (F1). The formulas for each metric are as follows:

(4) $$Acc=\frac{TP\;+\;TN}{TP\;+\;\;TN\;+\;\;FP\;+\;\;FN}$$

(5) $$Precision=\frac{TP\;}{TP\;+\;\;FP}$$

(6) $$Recall=\frac{TP\;}{TP\;+\;\;FN}$$

(7) $$F1=\frac{2\times Precision\times Recall}{Precision\;+\;\;Recall}$$

(8) $$\kappa =\frac{Acc-Pe}{1-\;Pe}$$

where TP indicates true positive, TN represents true negative, FP indicates false positive, FN indicates false negative, and Pe is the hypothetical probability of agreement by chance.

## 3. Results

This chapter is divided into four sections. Section A, “Five-class Sleep Stage Classification”, discusses our approach to categorizing sleep into five stages using single- and multi-channel data. We detail the performance metrics, the iterative training process, confusion matrices, and hypnograms to illustrate the differences in performance across different channels of data. Section B, “Multi-class Sleep Stage Classification”, presents the results of two-class, three-class, and four-class sleep stage classifications using single- and multi-channel data. This section compares the performance of sleep staging across different numbers of classes. Section C, “Leave-one-subject-out cross-validation”, shows the impact of subject variability using single- and multi-channel data. Section D, “Comparison of classification models”, presents a comparative analysis of different classification models, providing a comprehensive evaluation of the performance between single- and multi-channel data and verifying the superiority of the models proposed in this study.

A. Five-class sleep stage classification

The sleep stages using five-class classification were designated as Wake, N1, N2, N3, and REM. The results obtained using single- and multi-channel data across different data sets are summarized in Table 3, which indicate that the method using single-channel EEG with dual-channel EOG outperformed that using single-channel EEG with single-channel EOG, which, in turn, outperformed the approaches using single-channel EEG or single-channel EOG alone.

To avoid any misunderstanding, it should be clarified that multi-channel status is determined based on the number of signal channels. For instance, a single EEG channel is regarded as single channel, while two EEG channels are considered multi-channel. It is important to note that when referring to a single EEG channel, it means a re-referenced channel such as C3-M2, rather than the original EEG channels like C3 and M2.

In the Sleep-SC data set, the performance of the Fpz-Cz channel in single-channel EEG was superior to that of the Pz-Oz channel. Consequently, when forming the multi-channel data configuration, the EEG signal was selected with the Fpz-Cz channel. The single-channel Fpz-Cz achieved an accuracy of 89.77% and a Cohen’s Kappa coefficient of 0.796, with F1 scores for each sleep stage as follows: Wake, 97.57; N1, 41.73; N2, 83.29; N3, 80.49; and REM, 71.67. For the multi-channel (Fpz-Cz + EOG) data, an accuracy of 90.56% and a Cohen’s Kappa coefficient of 0.812 were achieved, with the corresponding F1 scores of 97.80 (Wake), 46.55 (N1), 83.38 (N2), 80.62 (N3), and 76.97 (REM). Based on the Sleep-SC data set, the experimental findings underscore the superior performance of the sleep staging classification model using multi-channel data, compared to single-channel data across all the evaluated metrics. The significant improvement in F1 scores for N1 and REM stages upon the addition of EOG channels is particularly noteworthy.

In the APPLES data set, the performance of the C3-M2 channel in the single-channel EEG method outperformed that of the C4-M1 channel. Therefore, when forming the multi-channel data configuration, the EEG signal was selected with the C3-M2 channel. Specifically, the single-channel C3-M2 yielded an accuracy of 76.28% and a Cohen’s Kappa coefficient of 0.664, with F1 scores as follows: Wake, 88.37; N1, 46.07; N2, 82.18; N3, 44.59; and REM, 78.25. Additionally, the C3-M2 channel with EOG(R) resulted in an accuracy of 76.93% and a Cohen’s Kappa coefficient of 0.677, with F1 scores of 89.07 (Wake), 47.74 (N1), 81.71 (N2), 45.77 (N3), and 82.1 (REM). In contrast, the multi-channel (C3-M2 + EOG(R) + EOG(L)) data exhibited improved performance, with an accuracy of 77.44% and a Cohen’s Kappa coefficient of 0.684, achieving F1 scores of 89.31 (Wake), 49.30 (N1), 82.09 (N2), 44.69 (N3), and 82.35 (REM). The results indicate that the C3-M2 channel with dual-channel EOG performed better than the C3-M2 channel with EOG(R) which, in turn, outperformed the single-channel approach.

In the SHHS1 data set, for the same reason, the C4-M1 channel was chosen in the multi-channel data configuration. Specifically, the single-channel C4-M1 achieved an accuracy of 79.64% and a Cohen’s Kappa coefficient of 0.714, with F1 scores of 87.93 (Wake), 22.64 (N1), 81.83 (N2), 79.64 (N3), and 68.59 (REM). Additionally, the C4-M1 channel with EOG(L) attained an accuracy of 82.22% and a Cohen’s Kappa coefficient of 0.754, with F1 scores of 90.63 (Wake), 27.25 (N1), 82.23 (N2), 81.23 (N3), and 77.59 (REM). In contrast, the multi-channel (C4-M1 + EOG(R) + EOG(L)) data exhibited improved performance with an accuracy of 82.65% and a Cohen’s Kappa coefficient of 0.76, achieving F1 scores of 90.74, 30.82, 83.52, 82.11, and 78.76 for Wake, N1, N2, N3, REM, respectively. The results indicate that the C4-M1 channel with dual-channel EOG performed better than the C4-M1 channel with EOG (L) which, in turn, outperformed the single-channel alone.

In the MrOS1 data set, for the same reason, the C3-M2 channel was chosen in the multi-channel configuration. The single-channel C3-M2 yielded an accuracy of 85.25% and a Cohen’s Kappa coefficient of 0.779, with F1 scores as follows: Wake, 94.1; N1, 33.49; N2, 84.91; N3, 77.28; and REM, 76.48. The C3-M2 channel with EOG(R) resulted in an accuracy of 85.77% and a Cohen’s Kappa coefficient of 0.789, with F1 scores of 94.68 (Wake), 36.17 (N1), 85.34 (N2), 77.9 (N3), and 80.9 (REM). In contrast, the multi-channel model (C3-M2+EOG(R)+EOG(L)) exhibited improved performance, with an accuracy of 87.18% and a Cohen’s Kappa coefficient of 0.807, achieving F1 scores of 95.26, 37.40, 86.44, 77.85, and 82.42 for Wake, N1, N2, N3, and REM, respectively. The results indicate that the C3-M2 channel with dual-channel EOG performed better than the C3-M2 channel with EOG (R) which, in turn, outperformed the single-channel alone.

**Figure 3:**
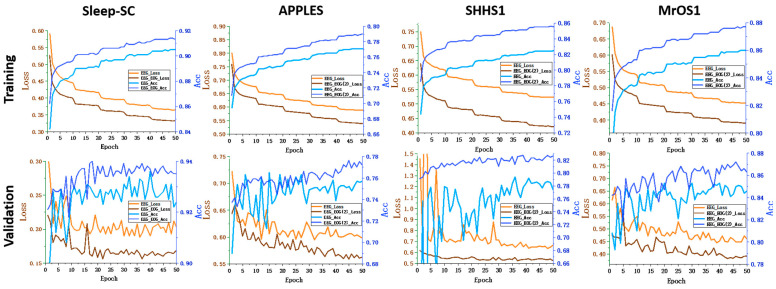
The training process using single- and multi-channel data over multiple epochs for a random fold (i.e., the first fold). Dark blue and light blue represent multi- and single-channel accuracy, respectively. Dark yellow and light yellow indicate multi- and single-channel loss, respectively. Multi-channel data consistently exhibit higher accuracy and lower loss than single-channel data across all data sets.

As shown in Figure 3, the training processes with single- and multi-channel data are illustrated over multiple epochs for a random fold (specifically, the first fold). Dark blue and light blue indicate the accuracy of the multi- and single-channel data, respectively; similarly, dark yellow and light yellow indicate the loss values using multi- and single-channel data, respectively. The results of all data sets demonstrate that multi-channel data consistently yielded higher accuracy and lower loss than single-channel data during the training process. Additionally, the convergence stability of multi-channel data is better than that of single-channel data. This improvement highlights the effectiveness of utilizing multi-channel data in enhancing the performance of sleep staging.

**Figure 4:**
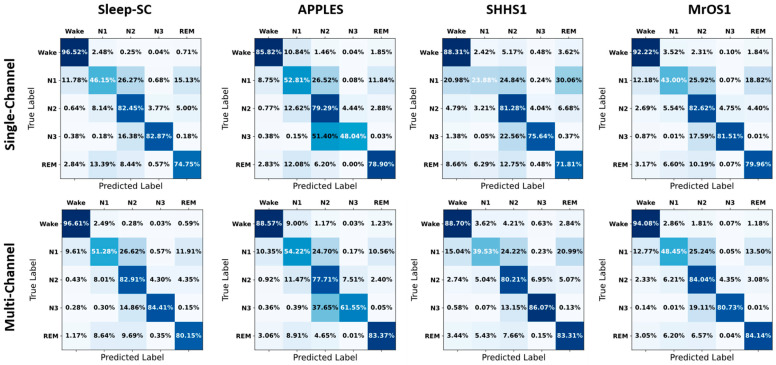
Confusion matrices for five-class sleep staging using single- and multi-channel data. For each data set, the performance of the model using multi-channel data is better than that using single-channel data, with particularly notable improvements in identification of the N1 and REM stages.

The confusion matrices in Figure 4 illustrate the true and predicted sleep staging classification values obtained with single- and multi-channel data. In detail, for the Wake stage, all data sets demonstrated the advantages of multi-channel data, with slight performance improvements. Specifically, the N1 stage—which is traditionally one of the most challenging sleep stages to accurately classify—exhibited marked improvements across all data sets when using multi-channel data; in particular, the accuracy of N1 classification increased by an average of 15–20% across the four data sets. Similarly, the REM stage classification exhibited a considerable enhancement when using multi-channel data, with an average improvement of 10–15% in accuracy. However, the N2 stage classification exhibited data set-dependent variations, with Sleep-SC and MrOS1 data sets demonstrating slight improvements in multi-channel data performance, while minor decreases were achieved when using the APPLES and SHHS1 data sets. Notably, in the latter two data sets, the misclassification of other stages as N2 was reduced, suggesting a potential trade-off in classification accuracy. For the N3 stage, although the MrOS1 data set resulted in a slight decrease in multi-channel data performance, the APPLES and SHHS1 data sets yielded significant improvements. Overall, across all data sets, the methods using multi-channel data consistently outperformed those using single-channel data, with particularly marked improvements in the classification of N1 and REM stages. This comprehensive analysis underscores the potential of multi-channel approaches in enhancing sleep stage classification performance, especially for stages that are traditionally more challenging to identify.

**Figure 5:**
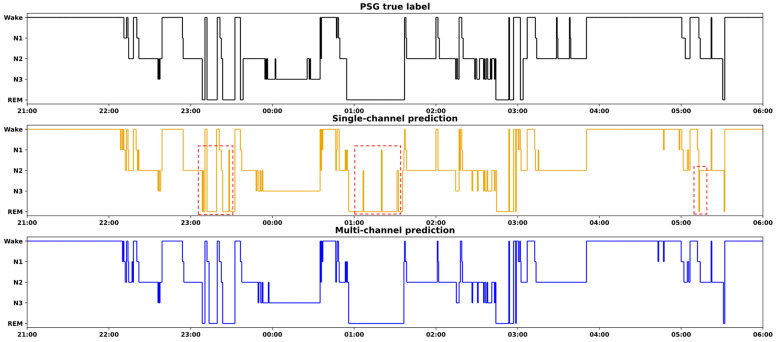
Examples of a hypnogram manually scored by sleep experts based on PSG data (**top**) and the hypnograms predicted with single-channel (**middle**) and multi-channel (**bottom**) data, using the MrOS1 data set. The red boxes indicate areas with potential misclassification errors using single-channel data, which were improved when using multi-channel data.

Figure 5 presents a comparative analysis of hypnograms for a subject from the MrOS1 data set. The top panel displays the hypnogram manually scored by sleep experts using PSG data. The middle and bottom panels illustrate the hypnograms predicted using single- and multi-channel data, respectively. Red boxes highlight areas where the single-channel predictions deviate from the expert-scored hypnogram, indicating a tendency toward misclassification errors. In contrast, the multi-channel hypnogram displays marked improvement in these same areas, as evidenced by closer alignment with the expert-scored version. This visual comparison effectively illustrates the enhanced accuracy of multi-channel data in capturing sleep stage transitions and maintaining consistency with expert annotations.

**B.**  **Multi-class sleep stage classification**

In practical applications, sleep staging requirements vary across different scenarios. To comprehensively evaluate the model’s performance using single- and multi-channel data in various tasks, additional experiments on sleep staging classification with 2–4 categories were conducted. Table 4, Table 5 and Table 6 present the results of the methods using two classes, (Wake and Sleep), three classes (Wake, NREM, and REM), and four classes (Wake, Light, Deep, and REM), respectively.

In the Sleep-EDF data set, the model using a combination of Fpz-Cz and EOG channels consistently outperformed other single-channel models, achieving the highest accuracy of 97.18% and a Cohen’s Kappa coefficient of 0.935 in the two-class method, 94.7% and 0.886 in the three-class classification, and 93.45% and 0.863 in the four-class classification. In the APPLES data set, the best performance was consistently obtained using the C3-M2 channel and dual-channel EOG, achieving an accuracy of 95.18% and a Cohen’s Kappa coefficient of 0.863 in the two-class classification, 90.88% and 0.833 in the three-class classification, and 87.14% and 0.778 in the four-class classification. In the SHHS1 data set, the combined use of the C4-M1 channel and dual-channel EOG yielded the highest accuracy of 94.09% and a Cohen’s Kappa coefficient of 0.862 in the two-class classification, 90.18% and 0.83 in the three-class classification, and 85.83% and 0.793 in the four-class classification. In the MrOS1 data set, the model using the C3-M2 channel and dual-channel EOG provided the best results, with an accuracy of 95.69% and a Cohen’s Kappa coefficient of 0.913 in the two-class classification, 92.93% and 0.88 in the three-class classification, and 90.2% and 0.846 in the four-class classification.

Additionally, in the APPLES, SHHS1, and MrOS1 data sets, the sleep staging classification performance of the model using a single EEG channel with a single EOG channel—although not reaching the level achieved with that using a single EEG channel with dual-channel EOG—consistently outperformed models with either a single EEG channel or a single EOG channel alone across all sleep staging classification tasks.

**Figure 6:**
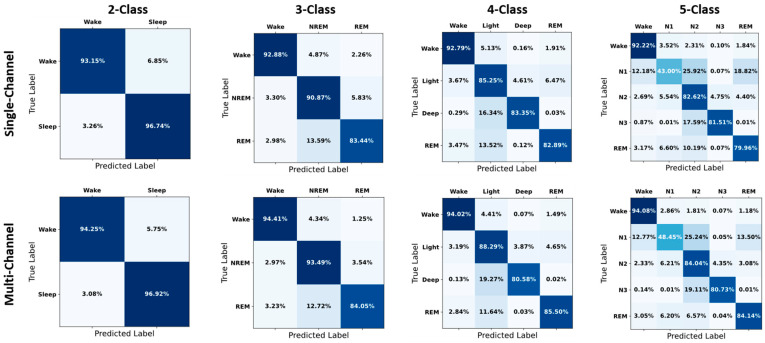
Confusion matrices for multi-class sleep staging with single- and multi-channel data on the MrOS1 data set. As the complexity of the classification tasks increases, the advantages of using multi-channel data become more evident, demonstrating improved accuracy and reliability in distinguishing between various classes. Light: Light sleep; Deep: Deep sleep.

These results also indicated a decline in classification performance with an increase in the number of classes. In Figure 6, taking the MrOS1 data set as an example, the confusion matrices provide a visual representation of the classification performance for single- and multi-channel models across all sleep staging classifications. The figure clearly illustrates the progressive increase in complexity and the corresponding decrease in overall accuracy as the number of classes increases. Moreover, while the Deep and N3 stages exhibited minor decreases in classification accuracy, the proportion of other stages being misclassified as Deep or N3 stages also decreased, suggesting a potential trade-off in classification accuracy.

In summary, the sleep staging results using 2–4 classes indicate that the method using single-channel EEG combined with dual-channel EOG consistently outperformed that using single-channel EEG with single-channel EOG which, in turn, outperformed the models using single-channel EEG or single-channel EOG alone. This conclusion is consistent with the results from the five-class sleep staging classification. Moreover, as the complexity of classification tasks increases, the advantages of utilizing multi-channel data become increasingly apparent, highlighting the improved accuracy and reliability when differentiating between various classes.

**C.**  **Leave-one-subject-out cross-validation**

**Figure 7:**
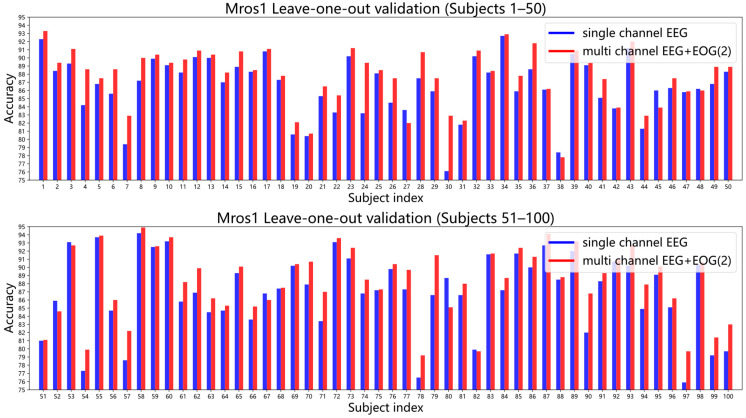
Comparison of five-class sleep staging accuracy for single-channel EEG and multi-channel data across 100 subjects from the MrOS1 data set. Single-channel EEG: C3-M2; Multi-channel EEG + EOG(2): C3-M2 + EOG(R) + EOG(L).

To comprehensively investigate the impact of single-channel versus multi-channel sleep staging, we examined the effect of subject variability using leave-one-subject-out cross-validation. To expedite the five-class sleep staging experiment, we selected the first 100 subjects from the MrOS1 data set. Each time, one subject was chosen as the test subject while the remaining 99 subjects were used for training. This process was repeated 100 times, yielding sleep staging results for both single-channel and multi-channel data for each subject. Detailed results are presented in Figure 7.

It can be observed that for the majority of subjects (91/100), multi-channel data outperforms single-channel data. Particularly, for some subjects, such as 7, 30, 54, 57, 78, 97 and 57, the improvement with multi-channel data is substantial. Interestingly, we found that for patients with poor single-channel sleep staging results, the use of multi-channel data significantly improved sleep staging performance. These leave-one-subject-out cross-validation results demonstrate that, despite significant individual differences in sleep staging, the conclusion that single-channel EEG combined with dual-channel EOG is more effective than single-channel EEG alone remains valid.

D. Comparison of classification models

In this section, we conducted a comparative analysis of the performance of various classification models for five-class sleep staging using both single- and multi-channel data. The results, spanning four distinct data sets and encompassing five classification models, are presented in Table 7.

The findings revealed that our hybrid attention neural network consistently outperformed the SVM, RF, EEGNet, and a simplified version of our network that lacked feature optimization, across all data sets and channels. This superior performance is attributed to the hybrid attention neural network’s robust feature extraction and optimization capabilities. The network’s ability to capture complex nonlinear relationships within the data and to differentiate relevant features through the attention mechanism provides a more nuanced understanding of sleep stages.

While EEGNet, specifically tailored for EEG signal analysis, generally performed as expected by outperforming SVM and RF, there were notable exceptions. In certain specific scenarios, such as with single-channel data from the SHHS1 data set, EEGNet’s performance was unexpectedly poor. However, in multi-channel scenarios, EEGNet demonstrated consistently better performance. This underscores the advantage of multi-channel data, which can significantly enhance the model’s generalization ability.

These results also reinforce the universal benefit of multi-channel data in sleep staging. Although the degree of performance improvement with multi-channel data varies across different classification models, the use of multi-channel data consistently achieved higher accuracy and better classification performance, irrespective of the model employed. Particularly, the combination of EEG and EOG data proved more effective than using single-channel EEG or EOG alone, reinforcing the notion that multimodal data fusion improves the accuracy and reliability of sleep stage classification.

In conclusion, the comparative experimental results not only highlight the superiority of the hybrid attention neural network proposed in this study but also confirm that the advantages of multi-channel data in sleep staging are universal and not model-specific. These findings are pivotal for the development of more precise sleep monitoring technologies and for propelling sleep research forward through the application of deep learning and multi-channel data analysis.

## 4. Discussion

In this study, a comprehensive investigation was conducted to evaluate the differences between single- and multi-channel data in terms of their effectiveness for automatic sleep staging classification. The research approach involved three key aspects: first, four diverse data sets—namely, Sleep-SC, APPLES, SHHS1, and MrOS1—were analyzed, each with distinct characteristics, thus ensuring that generalizable and reliable conclusions were obtained. Second, experiments were performed across various multi-task settings using sleep staging classifications with 2–5 categories to explore potential variations in performance across different complexity levels. Third, a hybrid attention neural network was established to integrate information from different channels, fully leveraging the advantages of multi-channel data.

It is crucial to highlight the significance of the reported improvements. Research indicates that even among clinicians, the inter-scorer agreement for the same data is approximately 83% [43]. Given that our model was trained using clinician-provided annotations as the gold standard, a 2–3% improvement over a high baseline is substantial, suggesting our model’s performance is nearing that of clinical experts. Furthermore, the consistent 2–3% improvement across four different data sets underscores the stability and robustness of using multi-channel data. Specifically, the model using single-channel EEG combined with dual-channel EOG outperformed that using single-channel EEG with single-channel EOG, which, in turn, outperformed the models using single-channel EEG or single-channel EOG alone. Furthermore, as the complexity of the classification task increases, the benefits of employing multi-channel data become more pronounced.

This hierarchical performance pattern emphasizes the complementary nature of EEG and EOG signals in automatic sleep staging. The improved performance observed with dual-channel EOG is particularly relevant in detecting the REM and N1 stages. The REM stage is a crucial part of the sleep cycle, which is associated with dreaming, brain function restoration, and memory consolidation; thus, its accurate identification is vital for comprehensive sleep analysis and diagnosis of sleep disorders [44]. Dual-channel EOG could capture conjugate eye movements, as eye movements are typically synchronous between the left and right eyes. Thus, recording the EOG of both eyes provides a more accurate representation of these movements, leading to more precise sleep staging. The identification of the N1 stage was enhanced through the combined use of EEG and dual-channel EOG, as they provide complementary information: while EEG captures overall brain activity patterns associated with different sleep stages, EOG provides specific information about the characteristics of eye movements. The synergy between EEG and EOG signals allows for a more robust and accurate classification of sleep stages. In contrast, recording only a single-channel EOG enables the recording of some eye movement information, but the data may not fully reflect the synchronous movements of both eyes, thus impacting the accuracy of sleep staging. Additionally, single-channel EOG is more susceptible to interference from EMG signals or other noise, which further reduces the reliability of the data.

In addition, compared to the other data sets, the Sleep-SC data set consistently demonstrated superior performance in sleep staging classification across both single- and multi-channel data. This may be attributed to the fact that the PSG data in the Sleep-SC data set are derived from healthy subjects without medication, whereas the other three data sets predominantly contain PSG data from patients with sleep disorders or other diseases. Consequently, the data from the Sleep-SC data set have higher regularity and consistency, as they are all from healthy subjects. As such, they are more suitable for automatic sleep staging. On the other hand, in the five-class sleep staging classification experiments, the APPLES data set exhibited the lowest performance. As the PSG data in the APPLES data set were derived from patients with OSA, this observation can be largely ascribed to the poor classification accuracy of the N3 stage. The study by Lu et al. [45] has revealed that OSA can significantly impact the N3 stage, often leading to a reduction in the amount and quality of sleep in this stage. Thus, its inaccurate identification is likely due to two factors: the less distinctive features of N3 epochs in patients with OSA and the relatively limited number of N3 epochs in the data set.

The limitations of this study and potential future work should also be highlighted. The first limitation is that our study focused solely on EEG and EOG channels, and did not explore other PSG channels such as ECG and EMG. These channels may provide additional insights for sleep staging classification. As sleep stages are associated with the autonomic nervous system (ANS) [46], and ECG is closely related to the ANS [47] while being relatively simple to measure and less prone to interference, it holds promise for patient-led sleep stage scoring. Future research could investigate whether incorporating the ECG channel can enhance sleep staging performance.

The second limitation is that despite improvements in N1 recognition using multi-channel data, the obtained results were still not in perfect agreement with the experts’ scores. Because the N1 stage is a transitional phase between wakefulness and sleep, and it shares similar characteristics with the REM stage, it is difficult for even sleep experts to accurately distinguish it. Danker et al. [48] examined the inter-operator variability of human expert scorers and found that the consistency for scoring the N1 stage was the lowest among all sleep stages. Moreover, as human sleep consists of several stages that are unevenly distributed, the number of N1 stage epochs is typically significantly lower than that of the other four stages, resulting in relatively fewer samples for model training. Despite the considerable difficulty in accurately identifying the N1 stage, future research should aim to further improve N1 detection accuracy. A promising method involves using discrete wavelet transform (DWT) to decompose EEG signals into five sub-bands. From each sub-band, four features are extracted: Higuchi’s fractal dimension, Katz’s fractal dimension, bubble entropy, and dispersion entropy. This results in a feature vector with 20 measures, enhancing the ability to distinguish between wakefulness and the N1 stage [49].

The third limitation is that in the data preprocessing section, only a band-pass filter and notch filter were used to remove basic noise. However, this approach may not adequately remove certain motion artifacts and external noise, such as electrical shifts and linear trends, which could interfere with the classification model’s training and prediction, reducing sleep staging performance. Future research should focus more on data preprocessing. An effective algorithm leveraging Stationary Wavelet Transform (SWT) and a kurtosis-based strategy was proposed to eliminate electrical shifts and linear trend artifacts (ESLT) from EEG signals [50]. Similarly, Noorbasha et al. [51] utilized Singular Spectrum Analysis (SSA) and Enhanced local Polynomial Approximation-based Total Variation (EPATV) filtering to remove these artifacts.

## 5. Conclusions

This comprehensive study investigated the effectiveness of single- versus multi-channel data in automatic sleep staging using a hybrid attention neural network across four distinct data sets and multiple classification tasks. The research yielded several important conclusions:

(1) Four public data sets with diverse characteristics (Sleep-SC, APPLES, SHHS1, and MrOS1) were used, covering a wide range of scenarios including healthy individuals, patients with OSA, and subjects with other conditions. This extensive validation ensures the broad applicability and reliability of our conclusions, rather than being based on a single or limited data set. Demonstrating the superiority of multi-channel data for automatic sleep staging consistently across different types of data sets provides more compelling evidence.
(2) By conducting experiments across various task settings, including 2-class, 3-class, 4-class, and 5-class classification, we comprehensively assessed the performance of our model under different levels of task complexity. Our findings indicate that the advantage of multi-channel data becomes more pronounced as the classification task complexity increases. This provides important insights for selecting methods in different sleep staging scenarios and extends the understanding of the effectiveness of multi-channel data beyond a single classification standard.
(3) The hybrid attention neural network was demonstrated to effectively exploit and integrate information from multiple channels, showcasing its potential as a powerful tool for automatic sleep staging. Our experimental results show that this model significantly outperforms other comparison models (such as SVM, RF, EEGNet, and simplified versions of the network) when handling multi-channel data and providing new directions for model improvement in the field of automatic sleep staging.
(4) The findings of our study have important implications for the development of portable sleep monitoring devices. Our analysis of the complementary nature of EEG and EOG signals in sleep staging shows that dual EOG channels significantly aid in detecting REM and N1 stages. Combining a single-channel EEG and dual-channel EOG sensors can enhance the accuracy and reliability of portable devices without significantly increasing their complexity, advancing sleep monitoring technology to meet the needs of both clinical practice and home health monitoring.

## Figures and Tables

**Table 1:** Descriptions of the four public evaluation data sets.

Data Set	Sleep-SC	APPLES	SHHS1	MrOS1
Subject composition	Healthy adults without medication	Most patients with OSA	Most subjects had coronary heart disease, stroke, or hypertension	Most subjects had diseases such as sleep disorders or angiopathy
Subject numbers	78	1516	6441	5994
PSG numbers	153	1104	5681	2907
Sample Rate	100 Hz	100 Hz	125/50 Hz	512 Hz
Age Distribution	25–101	18–84	40+	65+
Male/Female	37:41	509:279	1:1	All male

**Notation**: OSA: obstructive sleep apnea; PSG: polysomnogram.

**Table 2:** Distribution of used epochs after data pre-processing.

Data Set	Sleep-SC	APPLES	SHHS1	MrOS1
PSG numbers	152	500	500	500
Wake epochs	283,835	118,749	142,176	302,128
N1 epochs	21,469	71,321	17,621	23,869
N2 epochs	68,633	223,714	206,338	218,978
N3 epochs	12,991	12,389	53,469	38,847
REM epochs	25,767	66,097	68,768	69,534
Total epochs	412,695	492,270	488,372	653,356

**Notation**: PSG: polysomnogram; N1: non-rapid eye movement stage I; N2: non-rapid eye movement stage II; N3: non-rapid eye movement stage III; REM: rapid eye movement.

**Table 3:** The five-class sleep staging results obtained with single- and multi-channel data.

Data Set	Channel	Overall Metrics		Per-Class F1 Score				
		Acc (%)	κ	Wake	N1	N2	N3	REM
Sleep-SC	Fpz-Cz	89.77	0.796	97.57	41.73	83.29	80.49	71.67
	Pz-Oz	87.54	0.745	96.58	31.90	79.55	69.96	61.89
	EOG	89.67	0.794	97.47	40.89	83.32	79.96	70.65
	**Fpz-Cz + EOG**	**90.56**	**0.812**	**97.80**	**46.55**	**83.38**	**80.62**	**76.97**
APPLES	C4-M1	74.64	0.649	87.62	45.62	79.87	**47.09**	78.35
	C3-M2	76.28	0.664	88.37	46.07	**82.18**	44.59	78.25
	EOG(R)	71.93	0.615	85.31	43.66	76.50	41.78	78.33
	EOG(L)	70.91	0.604	85.65	45.36	75.28	42.90	77.02
	C3-M2 + EOG(R)	76.93	0.677	89.07	47.74	81.71	45.77	82.10
	**C3-M2 + EOG(R) + EOG(L)**	**77.44**	**0.684**	**89.31**	**49.30**	82.09	44.69	**82.35**
SHHS1	C4-M1	79.64	0.714	87.93	22.64	81.83	79.64	68.59
	C3-M2	78.43	0.702	87.50	26.62	79.64	77.35	71.14
	EOG(R)	79.39	0.713	88.47	23.13	80.03	75.61	75.97
	EOG(L)	79.55	0.716	88.56	24.72	80.56	75.34	76.60
	C4-M1 + EOG(L)	82.22	0.754	90.63	27.25	82.23	81.23	77.59
	**C4-M1 + EOG(R) + EOG(L)**	**82.65**	**0.760**	**90.74**	**30.82**	**83.52**	**82.11**	**78.76**
MrOS1	C4-M1	83.02	0.749	93.34	32.24	82.43	73.76	73.96
	C3-M2	85.25	0.779	94.10	33.49	84.91	77.28	76.48
	EOG(R)	83.66	0.756	93.53	30.37	82.85	69.25	78.32
	EOG(L)	83.51	0.754	93.65	30.75	82.78	68.83	78.14
	C3-M2 + EOG(R)	85.77	0.789	94.68	36.17	85.34	**77.90**	80.90
	**C3-M2 + EOG(R) + EOG(L)**	**87.18**	**0.807**	**95.26**	**37.40**	**86.44**	77.85	**82.42**

**Notation**: Bold indicates the best performance for each data set. Acc: accuracy; κ: Cohen’s Kappa coefficient; EOG(R): right EOG; EOG(L): left EOG; N1: non-rapid eye movement stage I; N2: non-rapid eye movement stage II; N3: non-rapid eye movement stage III; REM: rapid eye movement.

**Table 4:** The two-class sleep staging results obtained with single- and multi-channel data.

Data Set	Channel	Overall Metrics		Per-Class F1 Score	
		Acc (%)	κ	Wake	Sleep
Sleep-SC	Fpz-Cz	96.97	0.930	97.79	95.20
	Pz-Oz	95.71	0.899	96.89	93.05
	EOG	96.96	0.929	97.78	95.17
	**Fpz-Cz + EOG**	**97.18**	**0.935**	**97.94**	**95.53**
APPLES	C4-M1	94.80	0.851	88.47	96.64
	C3-M2	94.92	0.856	88.88	96.71
	EOG(R)	93.14	0.805	84.91	95.56
	EOG(L)	93.64	0.821	86.27	95.86
	C3-M2 + EOG(R)	95.05	0.859	89.14	96.80
	**C3-M2 + EOG(R) + EOG(L)**	**95.18**	**0.863**	**89.45**	**96.88**
SHHS1	C4-M1	92.61	0.824	87.72	94.72
	C3-M2	92.22	0.817	87.35	94.39
	EOG(R)	92.65	0.825	87.68	94.77
	EOG(L)	92.72	0.827	87.89	94.79
	C4-M1 + EOG(L)	93.90	0.856	90.03	95.61
	**C4-M1 + EOG(R) + EOG(L)**	**94.09**	**0.862**	**90.44**	**95.72**
MrOS1	C4-M1	94.64	0.892	94.06	95.11
	C3-M2	95.08	0.901	94.59	95.49
	EOG(R)	94.43	0.888	93.92	94.86
	EOG(L)	94.59	0.891	94.07	95.02
	C3-M2 + EOG(R)	95.55	0.910	95.09	95.93
	**C3-M2 + EOG(R) + EOG(L)**	**95.69**	**0.913**	**95.28**	**96.04**

**Notation**: Bold indicates the best performance for each data set.

**Table 5:** The three-class sleep staging results obtained with single- and multi-channel data.

Data Set	Channel	Overall Metrics		Per-Class F1 Score		
		Acc (%)	κ	Wake	NREM	REM
Sleep-SC	Fpz-Cz	93.87	0.868	97.69	89.48	70.18
	Pz-Oz	91.92	0.825	96.76	86.11	63.28
	EOG	93.86	0.868	97.70	89.58	69.75
	**Fpz-Cz + EOG**	**94.70**	**0.886**	**97.87**	**90.54**	**76.96**
APPLES	C4-M1	89.17	0.802	88.36	92.11	78.93
	C3-M2	89.54	0.810	88.97	92.30	79.80
	EOG(R)	87.87	0.779	85.64	91.00	78.72
	EOG(L)	87.83	0.777	86.03	90.88	78.25
	C3-M2 + EOG(R)	90.79	0.830	89.42	93.22	**82.92**
	**C3-M2 + EOG(R) + EOG(L)**	**90.88**	**0.833**	**89.86**	**93.29**	82.80
SHHS1	C4-M1	86.82	0.771	87.88	90.05	70.05
	C3-M2	86.89	0.771	88.07	89.68	72.04
	EOG(R)	87.99	0.793	88.38	90.90	75.34
	EOG(L)	88.57	0.800	88.53	91.37	76.56
	C4-M1 + EOG(L)	89.79	0.824	90.52	92.11	78.85
	**C4-M1 + EOG(R) + EOG(L)**	**90.18**	**0.830**	**90.85**	**92.32**	**79.90**
MrOS1	C4-M1	90.27	0.836	94.11	90.42	74.48
	C3-M2	91.02	0.849	94.45	91.19	76.22
	EOG(R)	91.57	0.856	94.09	91.84	79.18
	EOG(L)	91.44	0.853	94.08	91.69	78.34
	C3-M2 + EOG(R)	92.55	0.874	95.24	92.68	80.67
	**C3-M2 + EOG(R) + EOG(L)**	**92.93**	**0.880**	**95.39**	**92.96**	**82.29**

**Notation**: Bold indicates the best performance for each data set.

**Table 6:** The four-class sleep staging results obtained with single- and multi-channel data.

Data Set	Channel	Overall Metrics		Per-Class F1 Score			
		Acc (%)	κ	Wake	Light Sleep	Deep Sleep	REM
Sleep-SC	Fpz-Cz	92.61	0.846	97.67	85.22	80.34	70.93
	Pz-Oz	90.32	0.795	96.86	80.23	70.98	63.25
	EOG	92.58	0.846	97.67	85.07	**81.05**	70.52
	**Fpz-Cz + EOG**	**93.45**	**0.863**	**97.90**	**86.36**	79.88	**77.08**
APPLES	C4-M1	85.38	0.752	88.56	88.38	42.23	78.67
	C3-M2	85.91	0.759	88.90	88.82	43.66	79.03
	EOG(R)	83.75	0.722	85.62	87.07	36.70	78.50
	EOG(L)	83.37	0.709	84.53	87.07	33.52	75.44
	C3-M2 + EOG(R)	86.89	0.776	89.44	89.48	44.41	**82.33**
	**C3-M2 + EOG(R) + EOG(L)**	**87.14**	**0.778**	**89.59**	**89.83**	**46.07**	81.90
SHHS1	C4-M1	82.38	0.745	88.49	82.04	80.79	70.60
	C3-M2	81.47	0.732	88.01	80.67	77.64	72.13
	EOG(R)	81.53	0.733	88.02	80.95	74.69	75.02
	EOG(L)	82.11	0.740	88.35	81.73	74.60	76.40
	C4-M1 + EOG(L)	85.21	0.786	**90.86**	84.45	81.72	78.39
	**C4-M1 + EOG(R) + EOG(L)**	**85.83**	**0.793**	90.75	**85.31**	**82.00**	**79.56**
MrOS1	C4-M1	87.08	0.798	93.84	84.80	73.73	74.95
	C3-M2	88.38	0.818	94.39	86.23	77.37	77.20
	EOG(R)	87.72	0.806	93.99	85.60	69.46	78.43
	EOG(L)	87.60	0.804	93.83	85.61	69.00	78.54
	C3-M2 + EOG(R)	89.76	0.840	95.14	88.00	77.56	80.30
	**C3-M2 + EOG(R) + EOG(L)**	**90.20**	**0.846**	**95.31**	**88.37**	**77.90**	**81.86**

**Notation**: Bold indicates the best performance for each data set.

**Table 7:** The five-class sleep staging results obtained with single- and multi-channel data using five different classification models.

Data Set	Channel	SVM		RF		EEGNet		Our Method (No Feature Optimization)		Our Method	
		Acc	κ	Acc	κ	Acc	κ	Acc	κ	Acc	κ
Sleep-SC	Fpz-Cz	78.58	0.564	81.79	0.594	87.67	0.754	88.27	0.769	89.77	0.796
	Pz-Oz	83.64	0.655	85.45	0.687	86.08	0.707	86.55	0.725	87.54	0.745
	EOG	78.59	0.564	81.73	0.593	87.68	0.746	88.03	0.765	89.67	0.794
	**Fpz-Cz + EOG**	**86.90**	**0.741**	**88.30**	**0.764**	**89.49**	**0.791**	**89.68**	**0.796**	**90.56**	**0.812**
APPLES	C4-M1	64.56	0.503	69.58	0.543	72.69	0.613	73.94	0.631	74.64	0.649
	C3-M2	66.17	0.526	70.61	0.560	75.00	0.644	74.57	0.644	76.28	0.664
	EOG(R)	61.07	0.459	66.14	0.491	68.87	0.559	71.05	0.592	71.93	0.615
	EOG(L)	61.84	0.469	66.65	0.499	71.18	0.583	69.62	0.573	70.91	0.604
	C3-M2 + EOG(R)	70.29	0.584	74.26	0.610	76.07	0.664	76.22	0.666	76.93	0.677
	**C3-M2 + EOG(R) + EOG(L)**	**71.49**	**0.601**	**75.74**	**0.636**	**77.02**	**0.673**	**76.74**	**0.672**	**77.44**	**0.684**
SHHS1	C4-M1	72.84	0.619	74.63	0.636	40.38	0.094	77.30	0.686	79.64	0.714
	C3-M2	71.75	0.603	74.16	0.627	41.67	0.052	75.60	0.660	78.43	0.702
	EOG(R)	64.85	0.505	67.55	0.526	40.04	0.067	75.63	0.661	79.39	0.713
	EOG(L)	64.40	0.500	67.39	0.524	50.24	0.207	76.48	0.669	79.55	0.716
	C4-M1 + EOG(L)	78.48	0.698	80.38	0.717	80.29	0.726	80.59	0.731	82.22	0.754
	**C4-M1 + EOG(R) + EOG(L)**	**79.27**	**0.710**	**81.19**	**0.734**	**81.78**	**0.745**	**80.92**	**0.737**	**82.65**	**0.760**
MrOS1	C4-M1	76.99	0.648	78.54	0.662	80.63	0.709	82.27	0.735	83.02	0.749
	C3-M2	78.10	0.665	79.63	0.681	81.63	0.726	82.84	0.745	85.25	0.779
	EOG(R)	74.69	0.608	75.20	0.606	64.25	0.452	82.08	0.729	83.66	0.756
	EOG(L)	74.32	0.605	75.01	0.603	60.98	0.335	81.64	0.722	83.51	0.754
	C3-M2 + EOG(R)	81.59	0.721	83.85	0.747	86.09	0.789	85.21	0.779	85.77	0.789
	**C3-M2 + EOG(R) + EOG(L)**	**82.14**	**0.730**	**84.29**	**0.754**	**86.41**	**0.796**	**85.81**	**0.787**	**87.18**	**0.807**

**Notation**: Bold indicates the best performance for each data set.

## Data Availability

The data used in this study are publicly available and can be accessed through the PhysioNet database (https://physionet.org, accessed on 14–24 November 2023) and the National Sleep Research Resource (NSRR) database (https://sleepdata.org, accessed on 5 November 2023). No new data were created during this study.
